# *Vibrio vulnificus* RtxA1 Toxin Expression Upon Contact With Host Cells Is RpoS-Dependent

**DOI:** 10.3389/fcimb.2018.00070

**Published:** 2018-03-15

**Authors:** Rui Hong Guo, Ju Young Lim, Duong Nu Tra My, Se Jin Jo, Jung Up Park, Joon Haeng Rhee, Young Ran Kim

**Affiliations:** ^1^College of Pharmacy and Research Institute of Drug Development, Chonnam National University, Gwangju, South Korea; ^2^Department of Molecular Medicine, Chonnam National University, Gwangju, South Korea; ^3^Department of Microbiology, Clinical Vaccine R&D Center, Chonnam National University Medical School, Gwangju, South Korea

**Keywords:** RpoS, RtxA1, RtxB1, *Vibrio vulnificus*, cytotoxicity, LD_50_

## Abstract

The expression of virulence genes in bacteria is known to be regulated by various environmental and host factors. *Vibrio vulnificus*, an estuarine bacterium, experiences a dramatic environmental change during its infection process. We reported that *V. vulnificus* RtxA1 toxin caused acute cell death only when close contact to host cells was allowed. A sigma factor RpoS is a very important regulator for the maximal survival of pathogens under stress conditions. Here, we studied the role of RpoS in *V. vulnificus* cytotoxicity and mouse lethality. The growth of *rpoS* mutant strain was comparable to that of wild-type in heart infusion (HI) media and DMEM with HeLa cell lysate. An *rpoS* mutation resulted in decreased cytotoxicity, which was restored by *in trans* complementation. Interestingly, host contact increased the expression and secretion of *V. vulnificus* RtxA1 toxin, which was decreased and delayed by the *rpoS* mutation. Transcription of the cytotoxic gene *rtxA1* and its transporter *rtxB1* was significantly increased after host factor contact, whereas the activity was decreased by the *rpoS* mutation. In contrast, the *rpoS* mutation showed no effect on the transcriptional activity of a cytolytic heamolysin gene (*vvhA*). Additionally, the LD_50_ of the *rpoS* mutant was 15-fold higher than that of the wild-type in specific pathogen-free CD-1 female mice. Taken together, these results show that RpoS regulates the expression of *V. vulnificus* RtxA1 toxin and its transporter upon host contact.

## Introduction

RpoS is the master regulator of the general stress response that protects many bacteria from various harmful environmental conditions (Cavaliere and Norel, 2016; Somorin et al., 2017). The expressions of some virulence factors are tightly connected by RpoS to general cellular stress responses (Dulebohn et al., 2017). RpoS is activated by different environmental stresses, such as nutrient starvation (Aldsworth et al., 1999), heat, acid or alkaline treatment (Bhagwat et al., 2006), oxidative stress (Gawande and Griffiths, 2005), ultraviolet irradiation, and osmotic stress (Berney et al., 2006). Under conditions of nutrient deprivation, or as cells enter the stationary phase, *Escherichia coli* and related bacteria increase the accumulation of RpoS (Battesti et al., 2011). Although RpoS was first identified as a growth phase-dependent regulator, it is now understood to be a central regulator in the exponential phase as well as in stress adaptation (Schellhorn, 2014). RpoS regulates various virulence factors in different pathogenic bacteria, such as elastase of *Vibrio vulnificus* (Hülsmann et al., 2003), and mucosal escape response and hemagglutinin of *V. cholerae* (Silva and Benitez, 2006). It was also reported that RpoS plays a significant role in the response of *V. vulnificus* to bile and in the adaptation to low salinity (Chen et al., 2010; Tan et al., 2010).

*V. vulnificus*, an opportunistic pathogen, can cause primary septicemia when contaminated shellfish are eaten raw by susceptible subjects with underlying hepatic diseases, a heavy alcohol drinking habit, or other immunocompromised conditions (Tan et al., 2010). Genotype and capsular polysaccharide significantly impact serum survivability and pathogenicity of *V. vulnificus* (Williams et al., 2014). In the infection process, *V. vulnificus* experiences a dramatic environmental change from the seawater to the human body. The three most representative cytotoxins of *V. vulnificus* are large multifunctional autoprocessing repeats in toxin (MARTX), elastolytic protease (VvpE), and cytolytic hemolysin (VvhA) (Kim et al., 2008). RtxA1 is a MARTX that plays an essential role in *V. vulnificus* infection (Jeong and Satchell, 2012; Kim et al., 2013; Ziolo et al., 2014). RtxA1 is a composite toxin comprised of N-terminal repeat-containing regions, C-terminal repeat-containing regions and effector domains (Kwak et al., 2011; Kim et al., 2015). The *rtx* locus consists of two divergently transcribed operons: *rtxHCA* and *rtxBDE* (Lee et al., 2007; Gavin and Satchell, 2015). The *rtxA1* gene encodes an RTX cytotoxin (4701 amino acids), *rtxC* encodes an RtxA activator and *rtxBDE* encode the transporter system (Chen et al., 2003; Kim et al., 2008; Li et al., 2008). The *rtxHCA* operon is positively regulated by HlyU (Liu et al., 2007, 2009; Li et al., 2011; Wang et al., 2015). Histone-like nucleoid structuring protein (H-NS) represses expression of *rtxA1* operon by direct binding to the upstream region, and that HlyU binds to an overlapping region to replace H-NS from its binding site (Liu and Crosa, 2012). H-NS was reported to regulate the *V. vulnificus* virulence factors including VvhA and VvpE and H-NS positively regulates *vvpE* expression through the increase of the *rpoS* mRNA level (Elgaml and Miyoshi, 2015).

Our previous studies have reported that RtxA1 toxin kills host cells only after close contact of the bacteria with host cells (Kim et al., 2008). Because RpoS is a very important regulator for the maximal survival of pathogens under stress conditions, we studied the role of RpoS in *V. vulnificus* cytotoxicity and mouse lethality. We found that an *rpoS* mutation resulted in decreased cytotoxicity, so we suppose whether RpoS could regulate the main virulence RtxA1 expression. In the present study, we investigated the role of RpoS in the regulation of *V. vulnificus* RtxA1 toxin.

## Materials and methods

### Bacterial strains

*V. vulnificus* strains were grown in heart infusion (HI) broth (Difco, Becton-Dickinson, Bedford, MA, USA) at 37°C in a shaking incubator. MO6-24/O, a clinical isolate of *V. vulnificus* (Reddy et al., 1992) has been used as a wild-type strain, and the complete genome sequence has been annotated (Park et al., 2011). *V. vulnificus* CMM744 strain is a deletion mutant of the *rtxA1* gene (Kim et al., 2008). A deletion mutant of sigma factor RpoS (σ^38^) was constructed in MO6-24/O using a counter-selection strategy and the suicide vector pKAS32. The polymerase chain reaction (PCR) was used to confirm the internal deletion within the *rpoS* gene composed of 2,693 nucleotides.

For complementation of the *rpoS* mutant, DNA fragment containing wild-type *rpoS* gene with respective promoter (~1.2 kb) was amplified using primers listed in Table 1 (*rpoS*-F-*EcoR*I and *rpoS*-R-*Pst*I). Amplified DNA fragments were digested with appropriate restriction enzymes and subcloned into the broad host range vector pLAFR3. The resulting plasmids were transferred into the *rpoS* deletion mutant by the triparental mating using a conjugative helper plasmid pRK2013. The transconjugants were screened on TCBS (Difco, Becton-Dickinson, Bedford, MA, USA) agar plates with tetracycline 2 μg/mL and confirmed by PCR.

**Table 1:** Primers used in RT-PCR analysis for *rpoS* complementation.

	**Primers**	**Sequences**
*rpoS* RT-PCR	*rpoS*-F	5′-TGAAGCCGCACGTAAACGTATG-3′
	*rpoS*-R	5′-ACGGATGGTTCGAGTCTGATTC-3′
*rpoS* complementation	*rpoS*-F-*Eco*RI	5′-CGGAATTCTTAAAGCTGGGCAACAAATAGC-3′
	*rpoS*-R-*Pst*I	5′-AAAACTGCAGTCAATCCATATCGATATCAAAC-3′
*16S rRNA*	*16S rRNA-*F	5′-GTTGTGAGGAAGGTGGTGTC-3′
	*16S rRNA-*R	5′-CCGGGCTTTCACATCTGAC-3′

### Cytotoxicity assay of *V. vulnificus* strains to host cells

HeLa cells (Korea Cell Line Bank, Seoul, Korea) were maintained in high glucose Dulbecco's modified Eagle's medium (DMEM; Welgene, Daegu, Korea) with 10% heat-inactivated fetal bovine serum (FBS; ThermoFisher Scientific, Waltham, MA, USA) in a 37°C incubator with 5% CO_2_. To prepare *V. vulnificus* cells used in this study, overnight cultures were diluted 200-fold in 0.9% NaCl HI broth and further cultured in a 37°C shaking incubator at 200 rpm for 4 h. Bacterial pellets were prepared by centrifugal washing with PBS. The cytotoxicity of the *V. vulnificus* strains toward HeLa cells was measured by using a CytoTox96 non-radioactive cytotoxicity assay kit (Promega, Madison, WI, USA), as described elsewhere (Na et al., 2011). HeLa cells were cultured into 48-well cell culture plates (5 × 10^4^ cells/well) overnight (SPL Life Sciences Co., Ltd., Gyeonggi-do, Korea) and were washed with serum-free DMEM. Bacterial cells were infected to the HeLa cells at a multiplicity of infection (MOI) of 20 and then incubated in a 5% CO_2_ incubator at 37°C. Lactate dehydrogenase (LDH) released into the supernatant from HeLa cells was assayed as a marker of cytotoxicity according to the kit manufacturer's protocol.

### Growth of the *rpoS* mutant strain in DMEM with live HeLa cells

HeLa cells were cultured into 24-well cell culture plates (1 × 10^5^ cells/well) overnight (SPL Life Sciences Co., Ltd., Gyeonggi-do, Korea) and were washed with serum-free DMEM. Bacterial cells were then infected to the HeLa cells at an MOI of 20 and then incubated in a 5% CO_2_ incubator at 37°C for 120 min. *V. vulnificus* culture suspensions were 10-fold serially diluted with free DMEM, and then the serial dilutions (10 μL) were loaded on HI agar plates overnight.

### Staining of HeLa cells infected with *V. vulnificus* strains

Live cell images were obtained from HeLa cells cultured in 8-well-chambered coverslips of #1 German borosilicate (Nalge Nunc International, Rochester, NY, USA), as described previously (Kim et al., 2010). Bacterial cells were infected into the HeLa cells in serum-free DMEM at an MOI of 100 for 75 min. The HeLa cells were then incubated with Alexa Fluor 594-conjugated wheat germ agglutinin (WGA, red color) (ThermoFisher Scientific) for 10 min to visualize the cytoplasmic membranes. Fluorescence images were acquired using a laser scanning confocal microscope (Leica Microsystems TCS NT, Leica, Germany) at the Korea Basic Science Institute (KBSI, Gwangju, Korea).

### Swarming motility test

*V. vulnificus* wild-type or *rpoS* mutant strains were freshly grown on HI agar plates with 1.5% agar at 37°C. The bacteria were inoculated onto semisolid HI agar plates containing 0.3% agar and incubated for at 37°C for ~8 h, as previously described (Kim and Rhee, 2003).

### Adherence assay

HeLa cells were seeded into four-well Lab Tec chamber slides (Nunc, Naperville, IL, USA) and bacterial adhesion was assayed as previously reported (Kim and Rhee, 2003). Briefly, *V. vulnificus* wild-type or *rpoS* mutant strains were infected at an MOI of 250 for 30 min. HeLa cells were thoroughly washed three times with pre-warmed DMEM and stained with Giemsa solution (Merck, Darmstadt, Germany). Bacterial cells adhering to 90 HeLa cells were counted and the results reported as the average number of adhered bacteria per HeLa cell.

### Growth of *V. vulnificus* strains in serum-free DMEM or HeLa lysate media

To prepare HeLa lysate media, HeLa cells (5 × 10^6^ cells/dish) grown overnight in a 100-mm tissue culture dish were incubated in fresh serum-free DMEM for 90 min, harvested by scraping and sonication, and then sterilized through a 0.2-μM filter, as reported previously (Kim et al., 2003).

*V. vulnificus* wild-type or *rpoS* mutant strains were inoculated into DMEM or the HeLa lysate media at the concentration of 10^8^ CFU/mL and incubated in a 5% CO_2_ incubator at 37°C for 3 h. For enumeration of live bacterial cells, the *V. vulnificus* culture suspensions were 10-fold serially diluted with PBS, and the serial dilutions (10 μL) were loaded on HI agar plates overnight (Kim et al., 2010).

### Western blot analysis of RtxA1 protein

The RtxA1 protein was detected by Western blot analysis with antibodies specific to amino acids 1492–1970 (RtxA1-D2) or 4080–4701 (RtxA1-C), as described previously (Kim et al., 2013). HeLa cells (5 × 10^5^ cells/well) grown overnight in a 6-well plate (SPL Life Sciences Co., Ltd.) were washed with serum-free DMEM. Then, the cells were infected with *V. vulnificus* strains at an MOI of 20 at the given time intervals, and thereafter washed twice with cold DPBS (Welgene, Daegu, Korea). To prepare HeLa lysates, the cells were incubated with cell lysis buffer (Promega, Madison, WI, USA) supplemented with a protease inhibitor cocktail (Sigma-Aldrich, St. Louis, MO, USA) on ice for 30 min with gently shaking and then centrifuged at 13,000 rpm for 10 min. In addition, *V. vulnificus* wild-type or *rpoS* mutant strains were inoculated into the HeLa lysate media or DMEM (2 × 10^8^ CFU/mL) at the given time intervals, and bacterial pellets were collected. The culture supernatants of HeLa lysate media or DMEM infected with *V. vulnificus* (300 μL) were precipitated by adding 3-fold cold acetone.

The proteins used for Western blotting were quantified using Bradford's reagent (Bio-Rad, USA), and equal amounts of protein specimens were electrophoresed on 3–8% Tris-acetate gradient mini gels (ThermoFisher Scientific) and transferred onto a PVDF membrane (Millipore Ltd, Tullagreen, Carrigtwohill, Germany). After blocking, the membranes were incubated with appropriate primary antibodies at 4°C overnight and then horseradish-peroxidase-conjugated second antibodies. Immunoreactive proteins were visualized using an ECL Western blot detection system (Advansta, Menlo Park, CA, USA). For protein size estimation, a HiMark Pre-stained marker (ThermoFisher Scientific) was used.

### RNA purification and real-time PCR

Restoration of the *rpoS* gene expression in the *rpoS* complemented strain (*rpoS*^−^+pLAFR3::*rpoS*) was determined by conventional RT-PCR. Total RNAs from log-phase bacterial cells were isolated using an RNeasy minikit (QIAGEN GmbH, Hilden, Germany). One microgram of purified RNA was converted to cDNA using the QuantiTect® Reverse Transcription Kit (QIAGEN) according to manufacturer's protocol. The *16S rRNA* housekeeping gene was used as an internal standard. The fragments were resolved by electrophoresis on 1% agarose gel.

The transcriptional activities of the cytotoxic genes were evaluated by real-time PCR (qPCR). Forward and reverse primer pairs were designed as showed in Table 2. Primers for *recA* were used as an internal standard for the qPCR. *V. vulnificus* strains at 10^8^ CFU/mL were cultured in the HeLa lysate media, serum-free DMEM, or HI broth. Total RNAs from the bacterial cells were purified by using the NucleoZOL reagent (MACHEREY-NAGEL GmbH, Düren, Germany). First-strand cDNA was synthesized from 1 μg of DNase-treated RNA samples in a 20 μL reaction volume using the TOPscript™ RT DryMIX (dT18 plus) synthesis kit (Enzynomics, Daejeon, Korea). The qPCR assays were performed by using Rotor-Gene Q (QIAGEN GmbH, Hilden, Germany) and a qPCR 2× premix SYBR-Green with Low ROX (Enzynomics, Daejeon, Korea). The relative expression levels of *rtxA1, rtxB1, vvhA*, and *vvpE* genes were normalized to the expression of *recA* via the threshold cycle (ΔΔC_T_) (Livak and Schmittgen, 2001).

**Table 2:** Primers used in real-time PCR analysis.

**Gene**	**Primers**	**Sequences**
*recA*	*recA*-F	5′-GAC CAG TTG TTG GTA TCT CAG CC-3′
	*recA*-R	5′-CGA TTT CTG CCT TTG GCG TCA-3′
*rpoS*	*rpoS*-F	5′-GAC CAG TTG TTG GTA TCT CAG CC-3′
	*rpoS*-R	5′-CGA TTT CTG CCT TTG GCG TCA-3′
*rtxA1*	*rtxA1*-F	5′-CTG AAT ATG AGT GGG TGA CCT ACG-3′
	*rtxA1*-R	5'-TGC GGT TTG ATT TCA CCG C-3′
*rtxB1*	*rtxB1*-F	5′-CCA TTT TAG CAG AAG GCG GG-3′
	*rtxB1*-R	5′-GAT GTT GGC CAT GTT GGA TTG-3′
*vvhA*	*vvhA*-F	5′-CGG TAC AAT CGG CAA CGT CA-3′
	*vvhA*-R	5′-GGC GAA TGG ACC AAT GTA AGT GC-3′
*vvpE*	*vvpE*-F	5′-ATG GCA CAA GGT TTG GCA G-3′
	*vvpE*-R	5′-GTT GTT TGC GTC TAA ACG CAC-3′

### Lethality in mice caused by *V. vulnificus* strains

All animal procedures were conducted according to the guidelines of the Animal Care and Use Committee of Chonnam National University. The protocol was approved by the Committee. The bacterial suspensions in PBS were administrated to randomly bred specific pathogen-free CD-1 female mice (8 week-old) via intraperitoneal injection, as described elsewhere (Na et al., 2011). Five mice were tested for each group, and the infected mice were observed at 22°C animal room for 48 h. The LD_50_ values were calculated by the Kaplan-Meier survival analysis method using SPSS (Robinson, 2004).

### Statistical analysis

All results are presented as the means ± SD. Statistical comparisons were evaluated using student's *t-*test with a *P* < 0.05 considered statistically significant. All experiments were repeated at least three times, and the data were obtained at least in triplicate. The results shown are from representative experiments.

## Results

### *V. vulnificus-*induced cytotoxicity and morphological changes in HeLa cells were decreased by an *rpoS* mutation

**Figure 1:**
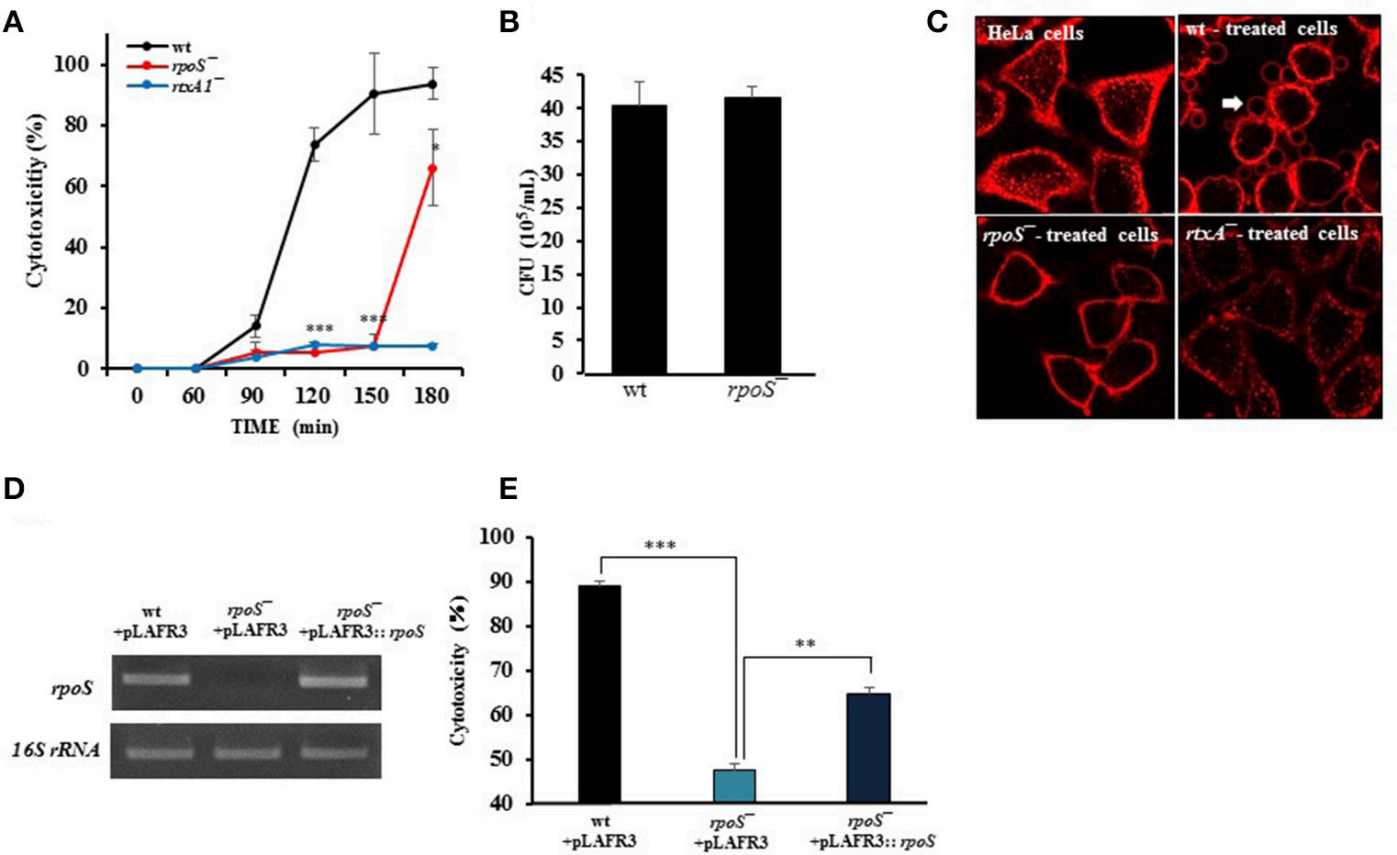
*V. vulnificus-*induced cytotoxicity and morphological changes in HeLa cells were decreased by an *rpoS* mutation. **(A)** HeLa cells in 48-well plates were infected with either *V. vulnificus* wild-type (wt), an *rpoS* mutant (*rpoS*^−^), or an *rtxA1* mutant (*rtxA1*^−^*)* strains at an MOI of 20. Lactate dehydrogenase released into the culture supernatant was assayed as a marker of cytotoxicity. **(B)** HeLa cells in 24-well plates were infected with *V. vulnificus* strains at an MOI 20 for 120 min. The bacterial suspension 10-fold serially diluted (10 μL) were loaded on HI agar plates overnight and the viable bacterial cells were counted. **(C)** HeLa cells cultured in an 8-well-chambered coverslip were infected with *V. vulnificus* strains at an MOI of 100 for 75 min. The cytoplasmic membranes were stained with Alexa Fluor 594-conjugated WGA (red color). **(D)** Restoration of the *rpoS* gene expression in the *rpoS* complemented strain (*rpoS*^−^+pLAFR3::*rpoS*) was determined by conventional RT-PCR. The *16S rRNA* housekeeping gene was used as an internal control. The fragments were resolved by electrophoresis on 1% agarose gel. **(E)** HeLa cells were infected with *V. vulnificus* strains with vector pLAFR3 at an MOI of 20 for 180 min, and the cytotoxicity was measured by LDH assay. Results represent the average of at least three independent experiments. Values are means ± SD (vs. *V. vulnificus* wild-type, ^**^P < 0.01, ^***^P < 0.001).

Live *V. vulnificus* is highly cytotoxic to host cells when the close contact is allowed (Kim et al., 2008). Hence, the role of a stress sigma factor RpoS on the cytotoxicity of the *V. vulnificus* was studied. First, the cytotoxicity to HeLa cells of *V. vulnificus rpoS* mutant was compared with a wild-type strain by using LDH assay. *V. vulnificus* wild-type strain caused a significant LDH release from 90 min (Figure 1A). By way of contrast, the cytotoxicity was delayed by the *rpoS* mutation. The *rtxA1* mutant showed dramatic reduced cytotoxicity toward HeLa cells as reported previously (Figure 1A). To ensure that the delayed cytotoxicity of *rpoS* mutant was not due to the decreased growth after host contact, we evaluated the growth of each strains in DMEM with live HeLa cells. Our results showed that *V. vulnificus rpoS* mutant strain showed the comparable growth with wild-type in live HeLa cells (Figure 1B).

To monitor the morphological changes, HeLa cells were stained with Alexa Fluor 594-conjugated WGA (red color). The *V. vulnificus* wild-type strain caused cell rounding, shrinkage, and blebbing of the HeLa cells, whereas the *rpoS* mutation dramatically decreased the morphological damage to the host cells (Figure 1C).

Also, we constructed three strains such as wt + pLAFR3, *rpoS*^−^+ pLAFR3, and *rpoS*^−^+ pLAFR3::*rpoS* to confirm RpoS complementation on the delayed cytotoxicity of *rpoS* mutant. The *rpoS* gene expression was detected by conventional RT-PCR. An *rpoS* complement strain showed the comparable *rpoS* mRNA level to the wild-type (Figure 1D). And the LDH assay also showed that the cytotoxic defect in an *rpoS* mutant strain was recovered in the complement strain *in trans* induced by the wild-type allele encoded by a plasmid (Figure 1E). These results show that RpoS protein has important roles in *V. vulnificus*-induced cytotoxicity and morphological change in HeLa cells.

### Comparable ability of wild-type and *rpoS* mutant strains on bacterial growth, motility, and adhesion

**Figure 2:**
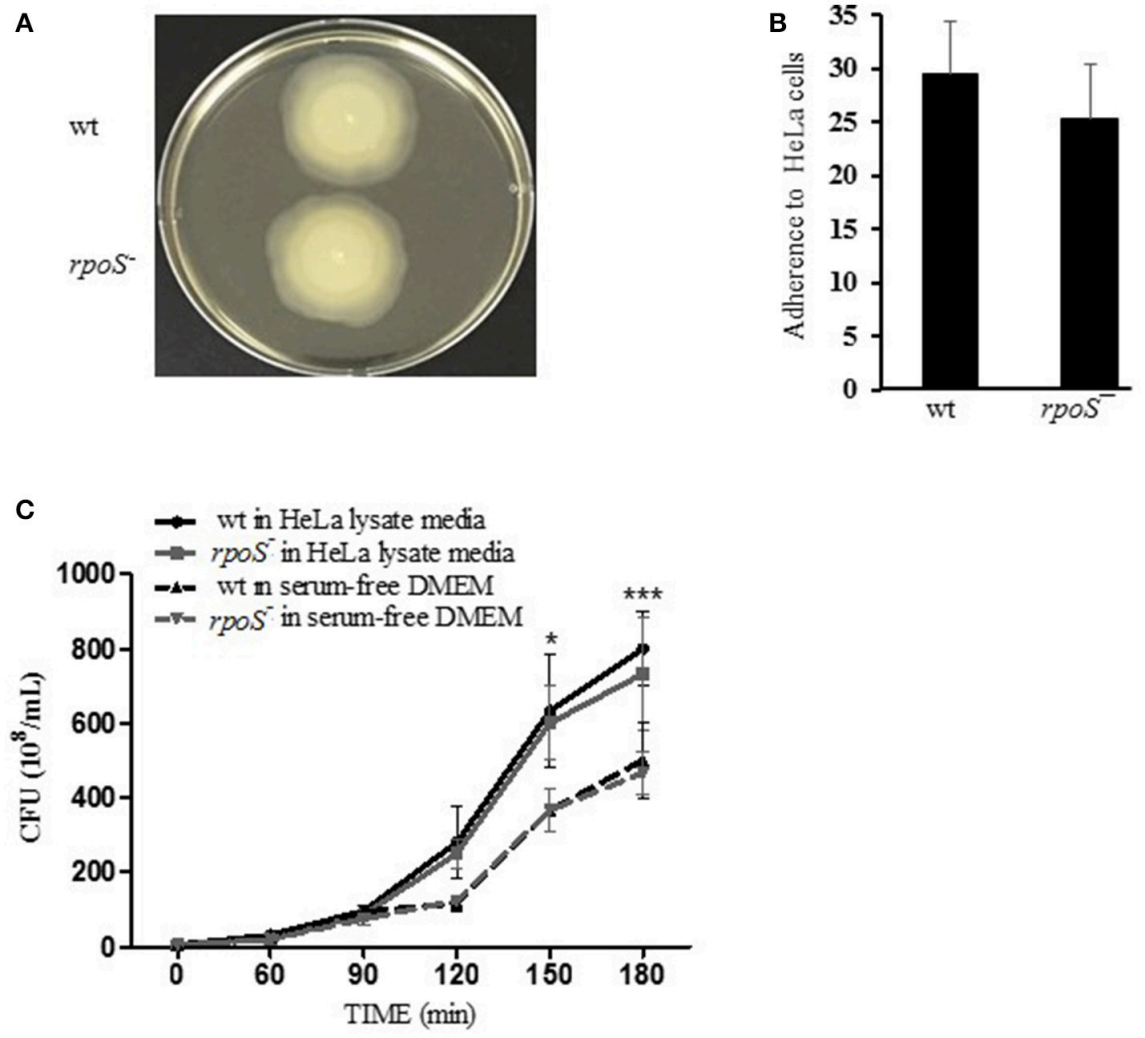
Comparable ability of wild-type and *rpoS* mutant strains on bacterial growth, motility, and adhesion. **(A)** To test swarming motility, the fresh cultured bacteria were inoculated by toothpicks on 0.3% semisolid HI agar plates, and incubated at 37°C incubator for 8 h. **(B)** To test bacteria adhesion, HeLa cells were incubated with *V. vulnificus* cells at an MOI of 250 for 30 min. The host cells were washed thoroughly and stained with Giemsa solution. The bacterial cells adhered to 90 HeLa cells were counted and calculated as the average number of adhered bacteria per HeLa cell. **(C**) To test bacterial growth, *V. vulnificus* wild-type or *rpoS* mutant bacteria were inoculated into DMEM or the HeLa lysate media (10^8^ CFU/mL) and incubated in a 5% CO_2_ incubator at 37°C for 3 h. The *V. vulnificus* culture suspensions were 10-fold serially diluted with PBS, and the serial dilutions (10 μL) were loaded on HI agar plates overnight. The results represent are means ± SD (vs. *V. vulnificus* wild-type in free DMEM, ^*^P < 0.05, ^***^P < 0.001).

Prior to studying the effects of *rpoS* mutant on *V. vulnificus* major virulence RtxA1, we first tested the ability of wild-type or *rpoS* mutant strains on motility and adhesion to live HeLa cells. The *rpoS* mutant strain did not show any differences with wild-type strain in motility and adhesion (Figures 2A,B).

The growth of *V. vulnificus* wild-type and *rpoS* mutant strains was also tested either in fresh DMEM or HeLa lysate media. Significant difference in growth was not observed between the isogenic wild-type and the *rpoS* mutant strains, and the bacterial growth in HeLa lysate media showed significant increase than that in free DMEM (Figure 2C).

### Host contact-induced RtxA1 expression was decreased by an *rpoS* mutation

**Figure 3:**
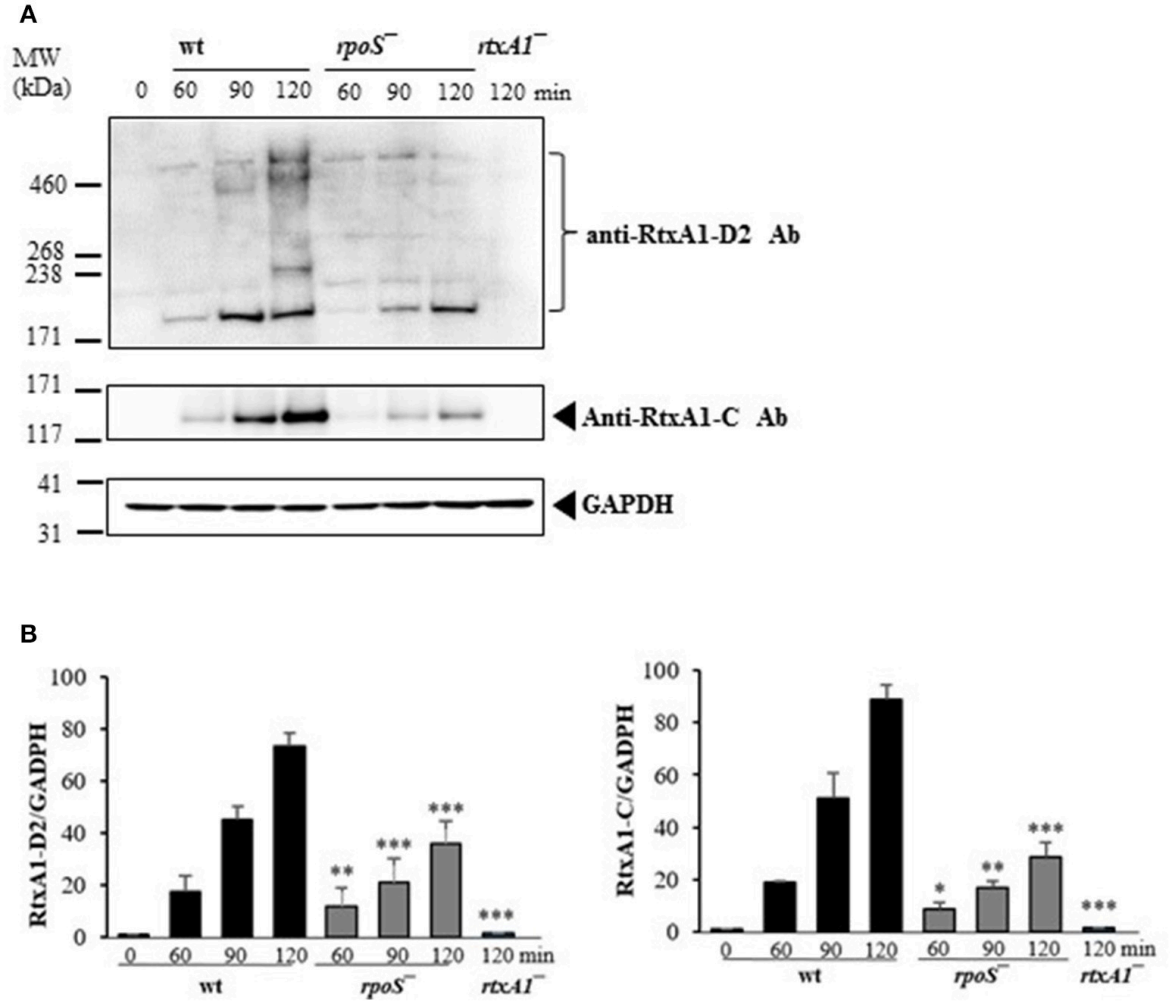
Host contact-induced RtxA1 expression was decreased by an *rpoS* mutation. **(A)** HeLa cells were infected with either *V. vulnificus* wild-type, an *rpoS* mutant, or *rtxA1* mutant strains at an MOI of 20. RtxA1 proteins in the HeLa lysates were detected by Western blot analysis. Two RtxA1 antibodies (Ab) specific to amino acids 1492–1970 (RtxA1-D2 domain) or 4080–4701 (RtxA1-C) were used. GAPDH was used as a loading control of HeLa lysates. The Western botting was conducted three times and the result shown is a representative experiment. **(B)** Protein levels from three experiments were analyzed and quantified using Image J software. Values are means ± SD (vs. *V. vulnificus* wild-type, ^*^P < 0.05, ^**^P < 0.01, ^***^P < 0.001).

The most representative cytotoxin of *V. vulnificus* is a multifunctional RtxA1 causing acute cell death. *V. vulnificus* RtxA1 causes host cell rounding and contact-dependent cytotoxicity (Kim et al., 2010). Intimate contact of the pathogen to the host cells constitutes a prerequisite for RtxA1-mediated cytotoxicity (Kim et al., 2008). Large RtxA1 proteins (501 kDa) of *V. vunificus* MO6-24/O strain are autoprocessed into at least two polypeptides: a large N-terminal fragment (RtxA1-N, ~370 kDa) and a smaller C-terminal fragment (RtxA1-C, ~130 kDa), during or after its secretion into the culture supernatant (Kim et al., 2013). We studied whether *rpoS* has any roles on RtxA1 production by Western blot analysis. HeLa cells were infected with the *V. vulnificus* wild-type, *rpoS* mutant, or *rtxA1* mutant strains respectively, and RtxA1 proteins in HeLa cell lysates were monitored by Western blot analysis. Two RtxA1 antibodies used are specific to amino acids 1492–1970 (RtxA1-D2 domain) or 4080–4701 (RtxA1-C) (Kim et al., 2013). The RtxA1 proteins in HeLa cell lysates infected with a *V. vulnificus* wild-type strain were increased gradually after host contact, which was decreased by the *rpoS* mutation significantly (Figure 3). Our results showed that RpoS was responsible for the upregulation of RtxA1 expression after host contact.

### Host factor-induced RtxA1 secretion was decreased by an *rpoS* mutation

**Figure 4:**
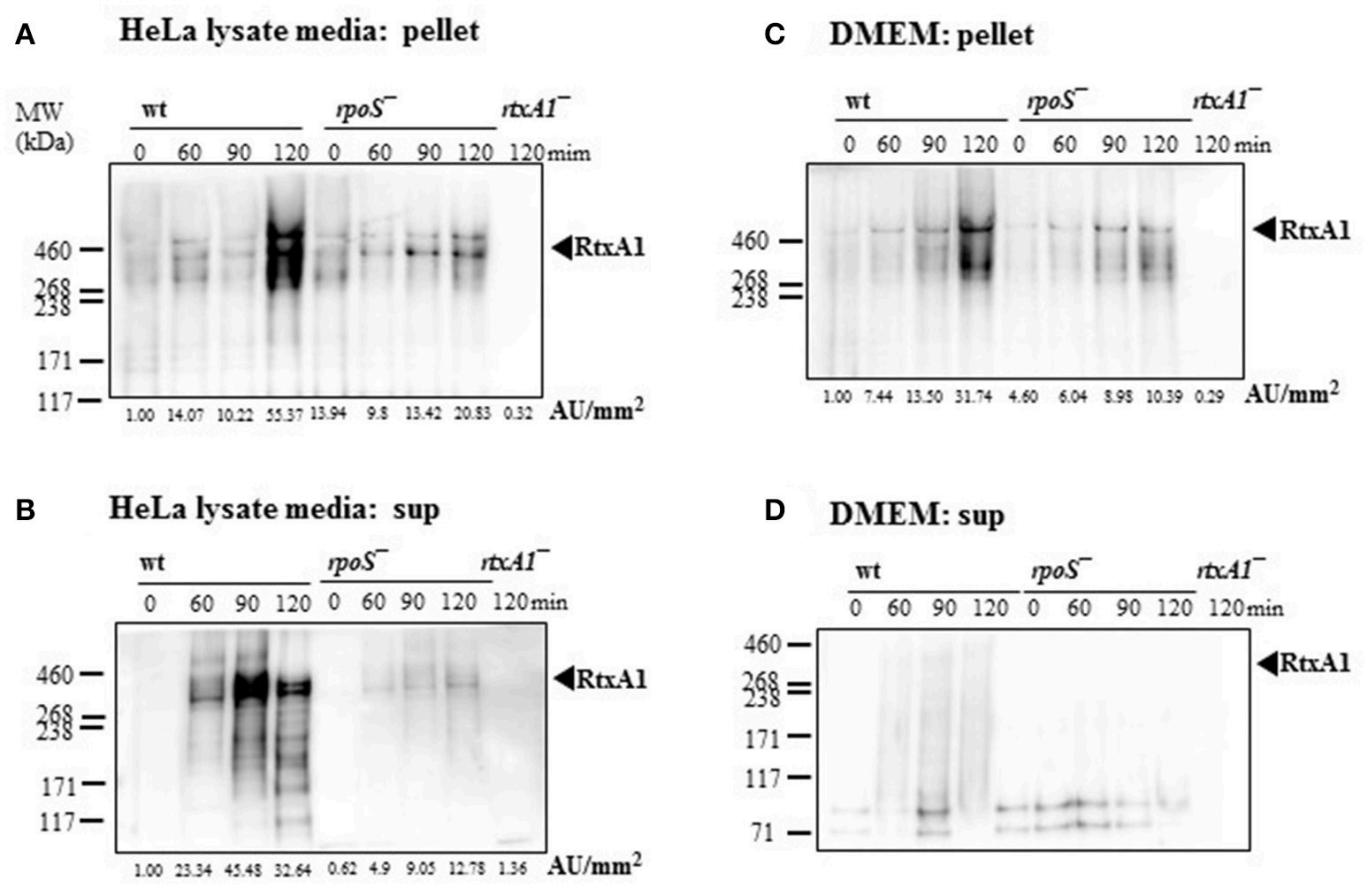
Host factor-induced RtxA1 secretion was decreased by an *rpoS* mutation. **(A)** *V. vulnificus* wild-type, an *rpoS* mutant, or *rtxA1* mutant strains at 2 × 10^8^ CFU/mL were incubated with HeLa lysate media in a 5% CO_2_ incubator at 37°C for the indicated times, and the pellets were collected for Western blot analysis. **(B)** RtxA1 Western blotting in the supernatant of bacteria-inoculated HeLa lysate media. **(C)** Bacterial cells at 2 × 10^8^ CFU/mL were incubated with DMEM in a 5% CO_2_ incubator at 37°C for the indicated times, and the pellets were collected for Western blot analysis. **(D)** RtxA1 Western blotting in the supernatant of bacteria-inoculated DMEM culture. RtxA1 toxin proteins were detected by Western blotting by using an anti-RtxA1 antibody specific to amino acids 1492–1970 (RtxA1-D2 domain). Protein levels were analyzed and quantified using Image J software. Abbreviation: AU, arbitrary units.

To investigate whether RtxA1 secretion after host contact is dependent to *rpoS* modulation, *V. vulnificus* strains (2 × 10^8^ CFU/mL) were incubated with either fresh DMEM without host factors or HeLa lysate media for 2 h. Western blot analysis of RtxA1 protein was conducted by using bacterial pellets and the culture supernatants. RtxA1 toxin was significantly increased in both *V. vulnificus* wild-type pellets and the supernatants incubated with HeLa lysate media (Figures 4A,B). The RtxA1 production in HeLa lysate media was more remarkable than that in fresh DMEM, and such increases were not shown in the *rpoS* mutation pellets (Figure 4). These results suggest that RpoS has a role in the expression and secretion of RtxA1 toxin.

### Transcriptions of cytotoxic genes in HeLa lysate media were regulated by an *rpoS* mutation

**Figure 5:**
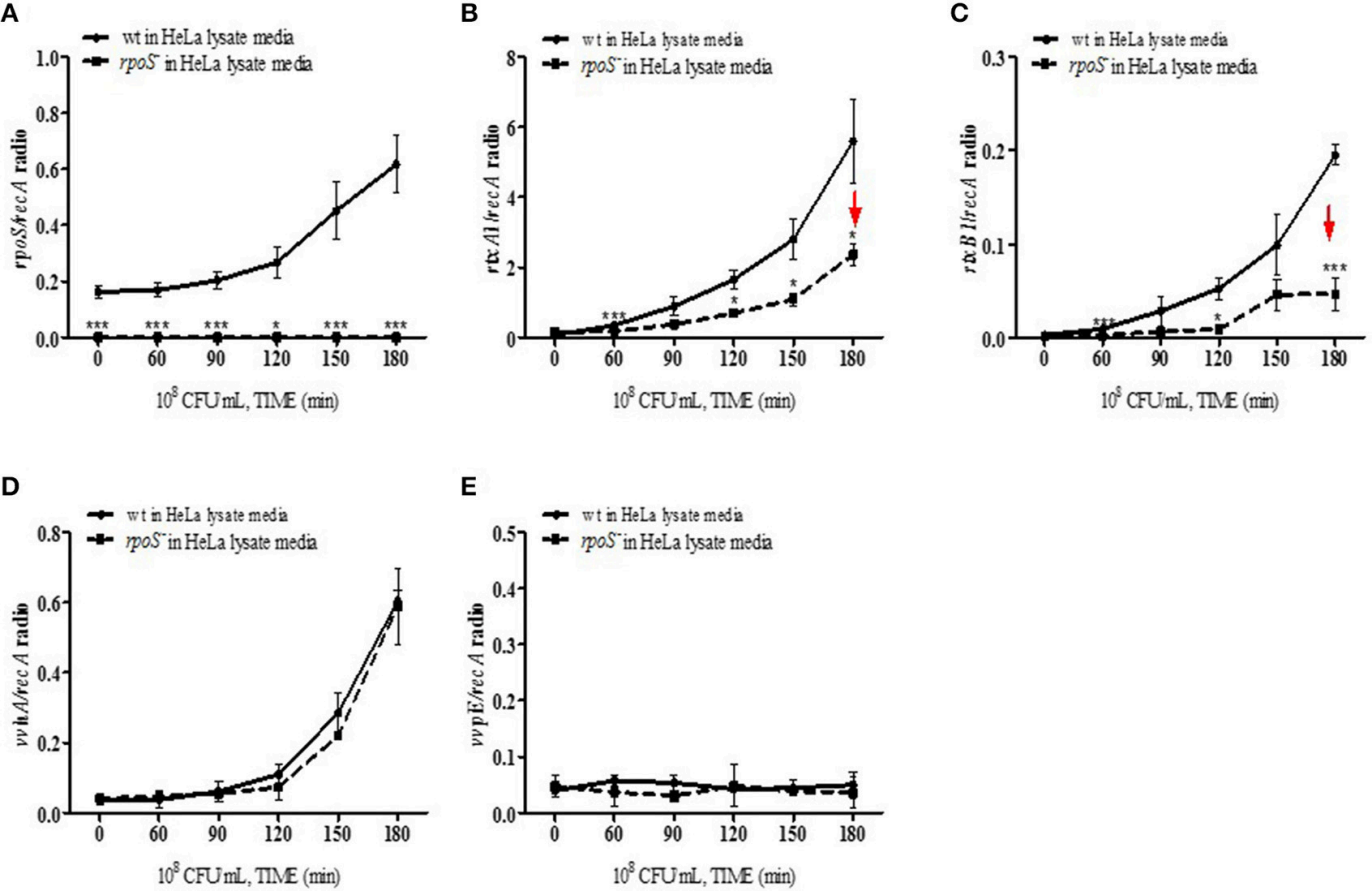
Transcriptions of cytotoxic genes in HeLa lysate media were regulated by an *rpoS* mutation. *V. vulnificus* wild-type or an *rpoS* mutant strains was inoculated into the HeLa lysate media, and the transcriptional activities of genes *rpoS* **(A)**, *rtxA1* **(B)**, *rtxB1* **(C)**, *vvhA* **(D)**, and *vvpE* **(E)** in each strains were separately estimated by qPCR. Results represent the average of at least three independent experiments. Values are means ± SD (vs. *V. vulnificus* wild-type, ^*^P < 0.05, ^**^P < 0.01, ^***^P < 0.001).

The effect of the *V. vulnificus rpoS* mutation on the transcriptional activities of the *rtxA1, rtxB1, vvhA*, and *vvpE* cytotoxic genes was examined by qPCR. *V. vulnificus* strains at 10^8^ CFU/mL were cultured in the HeLa lysate media and total RNAs were purified by using the NucleoZOL reagent. The transcriptional activities of *rpoS* gene was increased in *V. vulnificus* wild-type incubated with the HeLa lysate media (Figure 5A). The activities of *rtxA1* and *rtxB1* genes was increased in the wild-type strain, which was significantly reduced by the *rpoS* mutation (Figures 5B,C). The transcriptional activity of the *vvhA* was increased in an RpoS-independent manner (Figure 5D). However, there were no differences in the transcriptional levels of *vvpE* gene neither in the wild-type nor an *rpoS* mutant strains in HeLa lysate media throughout 3 h (Figure 5E).

### Mouse lethality caused by *V. vulnificus* was decreased by an *rpoS* mutation

**Figure 6:**
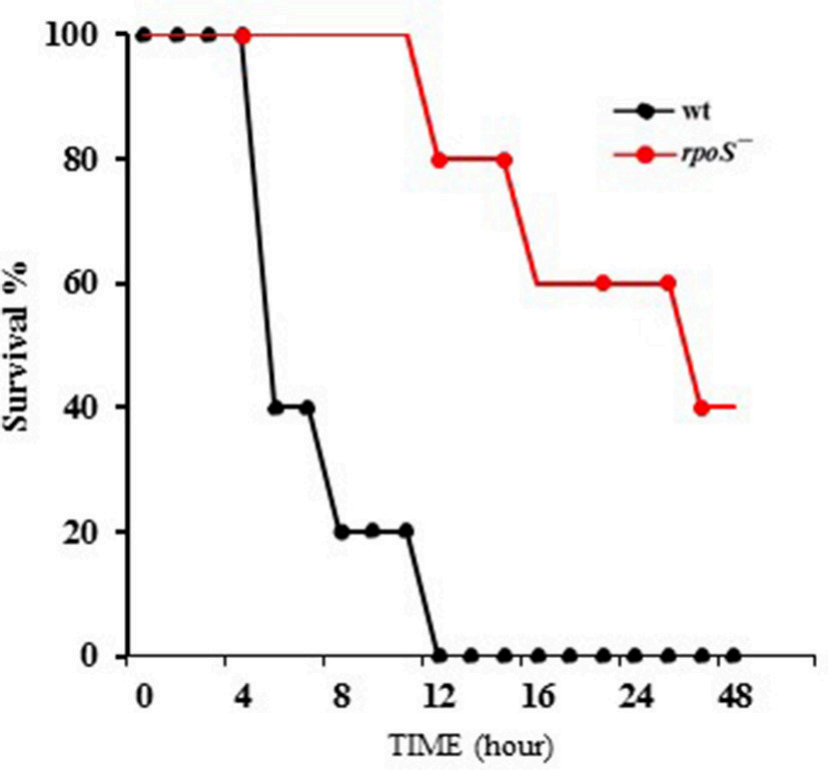
Mouse lethality caused by *V. vulnificus* was decreased by an *rpoS* mutation. *V. vulnificus* wild-type or an *rpoS* mutant strains (1 × 10^7^ CFU/mouse) were separately administered to 8 week-old CD-1 mice via the intraperitoneal route. Five mice were tested for each group, and the infected mice were observed for 48 h.

*V. vulnificus* virulence to mice was compared in wild-type and the *rpoS* mutant strains. The bacteria were administered to 8 week-old mice via the intraperitoneal route. The wild-type MO6-24/O-infected mice showed bristled fur, decreased activity, and acute lethality, whereas the *rpoS* mutant strain-infected mice showed ameliorated lethality (Figure 6). The *V. vulnificus rpoS* mutant exhibited a 15-fold increase in LD_50_ to mice relative to the wild-type (Table 3). These results showed that RpoS plays a decisive role in the pathogenesis of *V. vulnificus* infection.

**Table 3:** Effect of *rpoS* mutation on the LD_50_ of *V. vulnificus*.

**Strains**	**Relative genotype**	**LD_50_**
MO6-24/O	*V. vulnificus* wild-type, a clinical isolate	5.0 × 10^5^
*rpoS*^−^	MO6-24/O with a whole deletion of *rpoS* gene	7.3 × 10^6^

## Discussion

*V. vulnificus*, a halophilic estuarine bacterium, frequently causes a fatal septicemia with rapid progress, resulting in a mortality rate of more than 50% within a few days (Tacket et al., 1984). *V. vulnificus* encounters all kinds of environmental stress conditions during human infection (Bang and Drake, 2002). Thus, this bacterium is expected to use various efficient survival strategies to sense fluctuations in its surrounding conditions and to express the necessary defense elements against given stresses. Previous investigation had identified that RpoS was a key regulator mediating the survival of *V. vulnificus* (Park et al., 2004). The RpoS regulator, as an alternative sigma factor, controls the expression of many genes involved in the response to stress factors, as well as the transition of growth from the exponential to the stationary phase (Adnan et al., 2016). RpoS also plays an important role in biofilm development, bacterial virulence, persistence, and stress adaptation (Schellhorn, 2014).

In the present study, we constructed a *V. vulnificus rpoS* deletion mutant and tested for changes in its virulence phenotype. It is clear from our results in this work that RpoS is responsible for the cytotoxicity phenotype of this bacterium (Figure 1). In contrast, *V. vulnificus* wild-type and *rpoS* mutant strains showed the same growth either in live HeLa cells, HeLa lysate media or fresh DMEM (Figures 1B, 2C). Cytotoxic activity against HeLa cells was greatly delayed in an *rpoS* mutant, and the cytotoxic defect was recovered in part by the complementation *in trans* [*rpoS*^−^(pLAFR3:: *rpoS*)]. However, maybe due to a large vector pLAFR3 in size of about 25 kbp, the complementation did not show the equivalent cytotoxic with wild-type. *V. vulnificus* wild-type-induced host cell rounding and plasma membrane blebs was also decreased by the *rpoS* mutation (Figure 1C). Mice lethality was also decreased by an *rpoS* mutation (Table 3 and Figure 6), which indicates that *V. vulnificus* RpoS protein has very important roles during host infection process.

*V. vulnificus* shows acute cytotoxicity toward eukaryotic cells, and its RtxA1, a large pore-forming toxin causes host cell membrane permeabilization, cell rounding, and cell death (Lee et al., 2008; Liu et al., 2009). *V. vulnificus* MO6-24/O *rtxA1* gene encodes an RtxA1 protein with a molecular mass of 501 kDa, and the *rtxB1* with 701 amino acids serves as an ABC transporter system (Welch, 2001). To further determine the impact of *rpoS* mutation on the RtxA1 cytotoxicity, we measured the toxin production and gene expression in DMEM culture media with or without host factors. We found that *rpoS* mutation resulted in a decrease of host factor-induced RtxA1 production (Figures 3, 4A,B). In addition, the transcription of *rtxA1* and *rtxB1* genes was significantly increased in HeLa lysate media, which was dramatically reduced by the *rpoS* mutation (Figures 5B,C). However, there was no significant difference in *rtxA1* gene expressions in fresh DMEM without host factors (Figure S1), which was consistent with the results of RtxA1 production in DMEM (Figures 4C,D). Also, marginal increases of *rtxA1* and *rtxB1* gene expression were observed in HI broth, which were decreased by the *rpoS* mutation (Figure S2). These results suggest that the effect of RpoS on RtxA1 toxin expression is more significant upon host contact.

In contrast, the transcriptional activity of the hemolysin gene (*vvhA*) was not affected by the *rpoS* mutation (Figure 5D and Figure S2D). It is same as previous study reported that hemolysin activity was not affected by a mutation in *rpoS*, which encodes an alternative sigma factor, RpoS (σ^38^) (Choi et al., 2002). The transcriptional activity of protease gene (*vvpE*) showed no increase in the wild-type or the *rpoS* mutant cultured in HeLa lysate media used in this study throughout 3 h (Figure 5E). It was reported that the expression of the *vvpE* gene is dependent on RpoS factor in case of stationary phase growth in HI broth culture (Elgaml and Miyoshi, 2017). More *vvpE* expression was induced by the stationary-phase promoter dependent on RpoS (Jeong et al., 2003). We also found that the transcription of *vvpE* in the wild-type of *V. vulnificus* was increased in stationary-phase, which was significantly inhibited by the *rpoS* mutation in HI broth (Figure S2E).

The studies discussed in this work demonstrated that RpoS is an important regulator of the RtxA1 toxin and its transporter RtxB1 during infection. *V. vulnificus* RtxA1 toxin expression upon contact with host cells is RpoS-dependent. RpoS could bind directly to *rtxA1* promoter or regulate indirectly by modulation of H-NS and HlyU binding to the promoter. Cytosine nucleotide in the −35 region and −13C were noted in several sigma S-dependent promoters of *E. coli* (Wise et al., 1996; Landini et al., 2014). However, there are no the intrinsic promoter features supporting selective transcription by RpoS in *rtxA1* promoter legion reported by Crosa group (Liu et al., 2009). RpoS is tightly regulated at the transcriptional, translational, and posttranslational level. In stress condition of host contact or stationary phase, RpoS is increased and could play as a master sigma factor.

There exists an ample possibility that the RpoS-dependent RtxA1 regulation might be an indirect phenomenon. HlyU was reported to act as a derepressor of H-NS on the RtxA1 expression by direct binding to the promoter (Liu et al., 2009; Liu and Crosa, 2012). HlyU homodimer formation is required for binding to the upstream of *rtxA1* operon and is essential in relieving the H-NS repressor of rtxA1 transcription (Li et al., 2011). However, the HlyU binding site is exceptionally far from −35 and −10 sites where sigma factors bind. SmcR, a *V. harveyi* LuxR homolog was reported to repress the expression of hlyU by binding to a region upstream of the ORF (Shao et al., 2011). Indeed, transcriptional regulators (i.e., CRP), global regulators (IHF or Lrp), and nucleoproteins (such as H-NS or Fis) can influence sigma factor selectivity at various promoters (Landini et al., 2014). Previous study also analyzed the interaction between H-NS and RpoS in *V. cholerae*. H-NS could bind to *flrA* and *rpoN* promoters to repress their transcription, while RpoS acts to attenuate H-NS transcriptional silencing indirectly by enhancing the IHF expression which could compete with H-NS for binding to DNA and directly by promoting transcription initiation resistant to H-NS (Wang et al., 2012). H-NS was also reported to regulate positively vvpE expression through the increase of the *rpoS* mRNA level (Elgaml and Miyoshi, 2015). Taking above findings into account, there seems to be a rather complicated and intricate regulatory mechanism in the HlyU-SmcR-H-NS network in terms of virulence regulation related to RtxA1 expression.

To elucidate the mechanism by which the *rtxA1* promoter is activated by RpoS, the RpoS-binding sites at the promoter region should be mapped and their interaction with H-NS and HlyU needs to be investigated by using an *in vitro* transcription assay system. Subsequent genome-wide gene expression studies using gene arrays would elucidate the novel virulence genes under the control of the RpoS transcriptional regulator system.

## Conclusion

Our studies provide an insight that RpoS plays an important regulatory role in *V. vulnificus* cytotoxicity and infection. *V. vulnificus* RpoS modulates the expression and secretion of RtxA1 toxin through the transcriptional regulation of *rtxA1* and *rtxB1* genes in host-parasite interaction.

## Author contributions

RG, JL, DT, and SJ performed the experiments and wrote the manuscript. JP analyzed the data. JR provided the conceptual design. YK conceived the project, provided reagents, and the conceptual design and wrote the manuscript.

### Conflict of interest statement

The authors declare that the research was conducted in the absence of any commercial or financial relationships that could be construed as a potential conflict of interest.
