# Supplementary material for: Vibrio vulnificus RtxA1 Toxin Expression Upon Contact With Host Cells Is RpoS-Dependent
Source: Front Cell Infect Microbiol. 2018 Mar 15;8:70. doi: 10.3389/fcimb.2018.00070 (PMC5862816; doi:10.3389/fcimb.2018.00070)
Supplement: Supplementary file 1 [file DataSheet1.docx]

Fig.S1


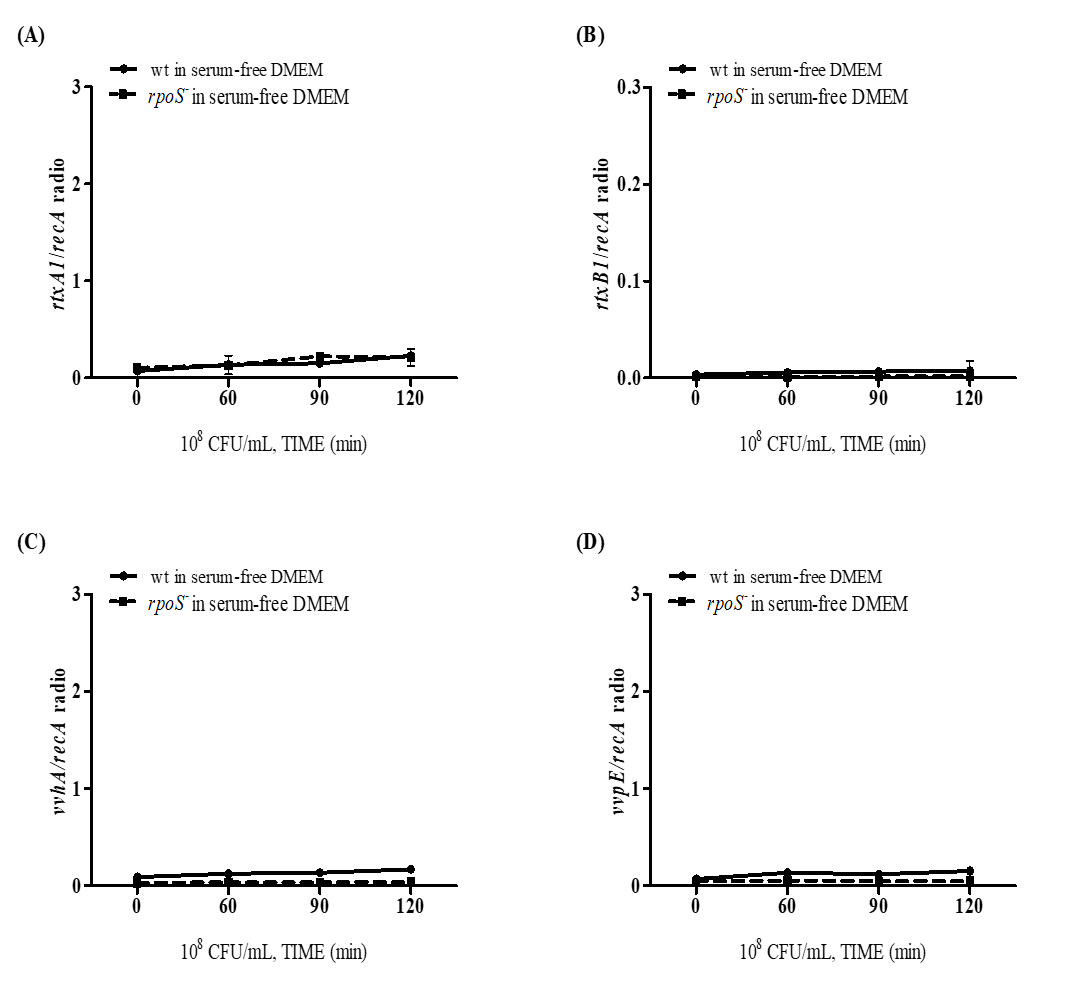


**Supplementary Figure S1. Regulations of *rpoS* mutation on the transcriptions of *rtxA1*, *rtxB1*, *vvhA*, and *vvpE* in fresh DMEM.** *V. vulnificus* wild-type or the *rpoS* mutant strains were cultured in DMEM in a 5 % CO_2_ incubator at 37 °C, and the expression of the *rtxA1* **(A)**, *rtxB1* **(B)**, *vvhA* **(C)**, and *vvpE* **(D)** genes in each strains was separately estimated by qPCR analysis. Data were normalized to the expression of the *recA* gene. Results represent the average of at least three independent experiments. Values are means ± SD.

Fig.S2


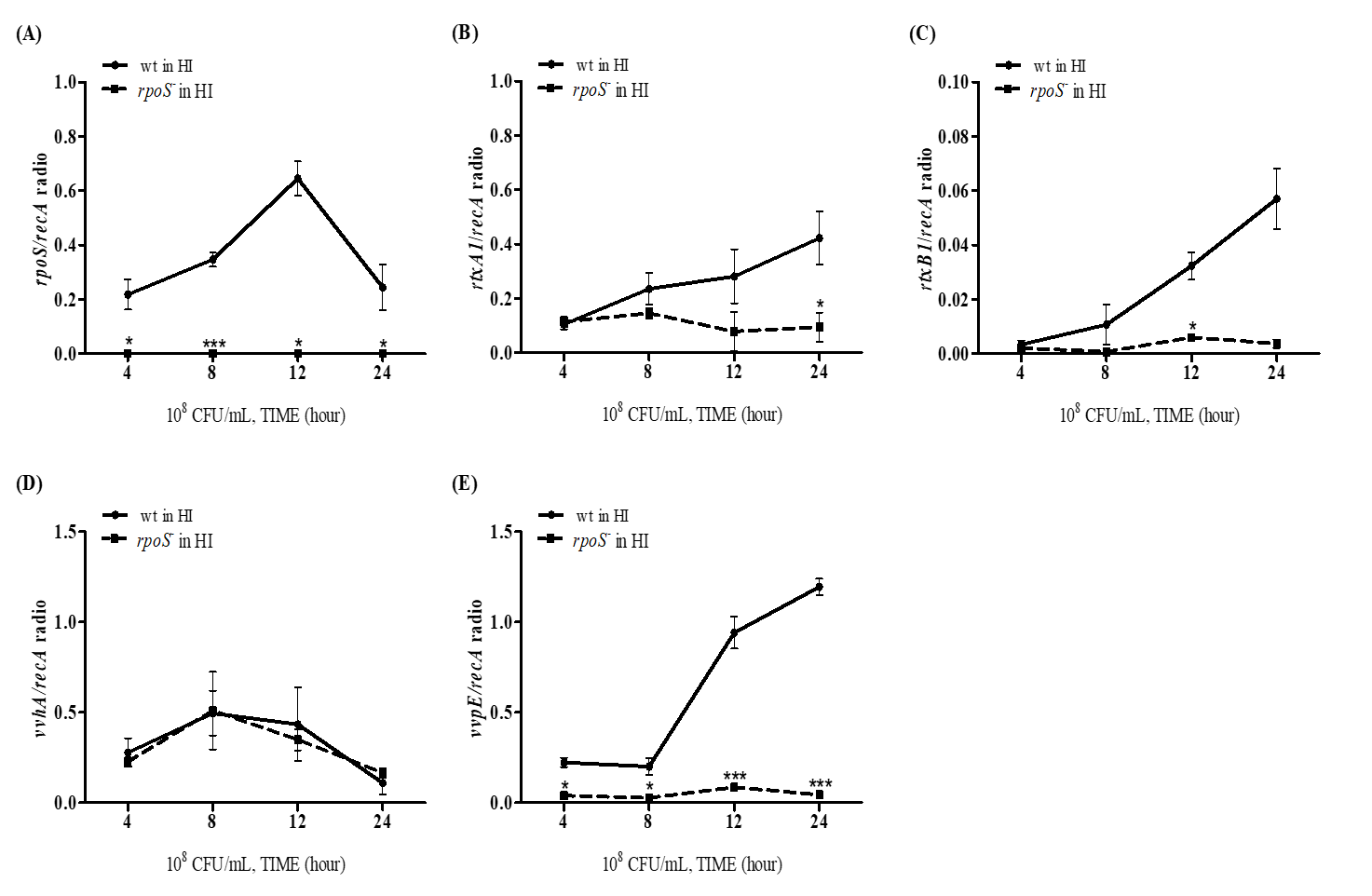


**Supplementary Figure S2. Regulations of *rpoS* mutation on the transcriptions of *rpoS*, *rtxA1*, *rtxB1*, *vvhA*, and *vvpE* in HI broth.** *V. vulnificus* wild-type or the *rpoS* mutant starins were cultured in 0.9 % NaCl-containing HI broth at 37 °C, and the expression of the *rpoS* **(A)**, *rtxA1* **(B)**, *rtxB1* **(C)**, *vvhA* **(D)**, and *vvpE* **(E)** genes in each strain was separately estimated by qPCR analysis. Data were normalized to the expression of the *recA* gene. Results represent the average of at least three independent experiments. Values are means ± SD (vs. *V. vulnificus* wild-type, *P < 0.05, ***P < 0.001).
